# Which ASDAS cut-off corresponds best to treatment intensification in patients with axial spondyloarthritis in daily practice? A prospective study from a clinical registry

**DOI:** 10.1007/s00296-025-06011-1

**Published:** 2025-10-23

**Authors:** Rabab Nezam El-Din, Astrid van Tubergen, Harald E. Vonkeman, Casper Webers

**Affiliations:** 1 Department of Rheumatology, Maastricht University Medical Centre, PO Box 5800, 6202 AZ Maastricht, The Netherlands; 2 Care and Public Health Research Institute (CAPHRI), Maastricht University, Maastricht, The Netherlands; 3 Department of Rheumatology and Clinical Immunology, Medisch Spectrum Twente, Enschede, The Netherlands; 4 Department of Psychology, Health & Technology, University of Twente, Enschede, The Netherlands

**Keywords:** Axial spondyloarthritis, Disease management, Registries

## Abstract

To investigate which Axial Spondyloarthritis Disease Activity Score (ASDAS) cut-off corresponds best with treatment intensification (TI) in daily practice in patients with axial spondyloarthritis (axSpA). Patients from the prospective SpA-Net registry with axSpA and ≥ 1 ASDAS measurement in 2016–2022 were included. TI was defined as (1) increasing dose/frequency of current drug, (2) switching drug(s) or (3) adding drug(s); all due to inefficacy of current treatment and only considering anti-inflammatory drugs. Patients could contribute multiple observations. Receiver operating characteristic analyses assessed the ability of ASDAS to discriminate between TI/non-TI (Area Under the Curve [AUC]), and identify the ASDAS cut-off that discriminated best. In a random subsample, the rationale for treatment decisions was retrospectively analyzed using patient records. In total, 350 patients with 2,191 ASDAS measurements (243 TI events, 11.1%) were included. Median follow-up was 2.8 years. At inclusion, mean age was 48.2 (SD 14.3) years, 152 (43.4%) were female, and mean ASDAS was 2.4 (SD 1.0). The mean ASDAS was 3.0 (SD 1.0) at TI versus 2.3 (SD 1.0) at non-TI timepoints. TI occurred infrequently at ASDAS ≥ 2.1 observations (203/1,266 [16.0%]). Using all observations, the AUC was 0.71 (95%CI 0.68–0.74) with an optimal ASDAS cut-off of 2.7 (sensitivity 69%, specificity 66%). When stratifying by drug exposure or extra-musculoskeletal manifestations, results were similar (ASDAS cut-off 2.6–3.2). The patient record analysis supported the findings. In daily practice, TI is associated with a higher ASDAS cut-off than the recommended one (≥ 2.1). Rheumatologists consider factors beyond disease activity when making treatment decisions.

**Supplementary Information:**

The online version contains supplementary material available at 10.1007/s00296-025-06011-1.

## Introduction

In the management of axial spondyloarthritis (axSpA), disease activity is an important outcome [1]. It is preferably measured with the Axial Spondyloarthritis Disease Activity Score (ASDAS) [2]. Various pharmacological treatments are available to reduce disease activity, including non-steroidal anti-Inflammatory drugs (NSAIDs), biological disease modifying anti-rheumatic drugs (bDMARDs), targeted synthetic DMARDs (tsDMARDs) and, in certain situations, conventional synthetic DMARDs (csDMARDs) and glucocorticoids [1, 3, 4].

Some recommendations advise to adopt a treat-to-target (T2T) strategy in axSpA, aiming for a state of remission (inactive disease [ID], ASDAS < 1.3) or, alternatively, low disease activity (LDA, ASDAS < 2.1) [1, 5]. In case this target is not achieved, intensification of the pharmacological treatment is recommended. Of note, not all management recommendations support the use of T2T strategies in axSpA because of limited evidence, and there is ongoing debate on the benefits of T2T in axSpA [6]. The only available T2T trial in axSpA (TICOSPA) failed to meet its primary endpoint, although secondary outcomes and the health-economic analysis favoured a T2T strategy over usual care [7]. A previous multicenter study from our research group highlighted a noteworthy disparity between recommendations and practice, wherein only one-fifth of patients with axSpA and an HDA state – thus failing to meet treatment targets – received treatment intensification [8]. We also showed that the decision to intensify treatment in patients with an HDA state is associated with physician-centered factors, such as the physician’s judgement of disease activity, but not with patient-centered factors or the ASDAS [9]. Likewise, another study showed that treatment adaptations were not steered by disease activity status (according to the Bath Ankylosing Spondylitis Disease Activity Index [BASDAI]), but by the physician’s opinion on disease activity status [10]. Together, these studies demonstrate a discordance between T2T recommendations for axSpA and the real-world clinical application of this strategy. One of the factors that contribute to this discordance might be the use of the ASDAS and its cut-off values, which have been originally developed and validated for research purposes, with confirmatory studies in both radiographic and non-radiographic axSpA, and in males and females [11–14]. In 2010, the optimal ASDAS cut-off value corresponding to initiation of tumor necrosis factor-alpha inhibitors (TNFi) was studied in patients with radiographic axSpA. Findings indicated that, depending on the ASDAS version utilized, this cut-off ranged between 2.4 and 3.3 [15]. However, this study was conducted in an era in which the ASDAS had recently been introduced. T2T had not been recommended yet in axSpA and only TNFi were considered for its development, while the treatment armamentarium for axSpA has expanded substantially nowadays [15]. The current role of the ASDAS in clinical practice has yet to be elucidated, and information regarding the ASDAS cut-off values associated with any form of pharmacological treatment intensification in current clinical care is lacking. Therefore, the objective of the present study is to examine which ASDAS cut-off values correspond with treatment intensification in axSpA in clinical practice. In this regard, it is important to emphasize that our goal was not to define new ASDAS cut-off values.

## Methods

### Study design and data collection

For this observational study, data were used from a disease-specific integrated electronic health system for management and routine monitoring of Dutch patients with SpA (SpA-Net) [16]. SpA-Net is registered in the Netherlands Trial Registry (NTR 6740) and was implemented in routine care in May 2016 in two large hospitals in the Netherlands: an academic centre (Maastricht University Medical Centre, MUMC+) and a general hospital (Medisch Spectrum Twente, MST).

SpA-Net contains data on patient-reported outcomes (PROs), such as disease activity, physical function and pain, collected through questionnaires completed at home. Additionally, physician-reported outcomes (such as joint/enthesitis counts), laboratory data (including C-reactive protein), and data on medication use and adverse events complete the registry. The data were prospectively collected before (PROs) or during (physician-reported outcomes, laboratory data, medication use) routine outpatient consultations. As SpA-Net was used as an electronic patient record for follow-up of patients in daily practice, the frequency and extent of data collection varied among patients.

The ethics committee of MUMC + determined that observational studies in SpA-Net do not require official approval, as data were collected in routine care and are not subject to the Medical Research Involving Human Subjects Act (METC azM/UM 15-4-266, date February 23, 2016). Written informed consent was obtained from each patient to use their data for research.

### Study population

Any patient with a clinical diagnosis of SpA was eligible for enrollment in SpA-Net. No other inclusion or exclusion criteria were applied, and both prevalent and incident diagnoses were eligible. For the current study, SpA-Net participants with a clinical diagnosis of axSpA, data on treatment and ≥ 1 ASDAS measurement during the study period were included. Each participant could contribute multiple observations. For the current study, data collected in the period of May 2016 to December 2022 were used.

### Disease activity

ASDAS scores were calculated automatically by the system at each outpatient consultation based on PRO components as completed by the patient and CRP values entered by the rheumatologist, and visually presented to both the rheumatologist and the patient [16].

### Outcome

The outcome of the current study was treatment intensification (TI). TI was defined as (1) a higher dose or frequency of the same drug, (2) a switch of the current drug to another drug, or (3) the addition of new drug to the current treatment regime, all due to inefficacy of the current treatment regime. Only anti-inflammatory medication classes were considered for TI: NSAIDs, csDMARDs, bDMARDs, tsDMARDs and glucocorticoids.

Medication use was updated in SpA-Net by the treating rheumatologist at each outpatient consultation, as part of routine care. Of note, in practice there could be a slight delay between the ASDAS date and the accompanying change in treatment, for example because screening for latent infections is required before starting a bDMARD. For this reason, treatment changes up to 6 weeks after an ASDAS observation were still considered for the outcome.

### Manual check of reasons for non-TI

The rationale behind treatment decisions was not collected in a standardised manner in SpA-Net. In order to facilitate interpretation of results, the reason for non-TI at time of ASDAS ≥ 2.1 was retrospectively assessed in a random subsample (10% of total study population) of participants from the MUMC+, using the free-text electronic patient records in SpA-Net.

### Statistical analysis

Patient and disease characteristics were described at time of inclusion in the current study. Furthermore, the number and characteristics of TI events were described. In the random subsample of those with ASDAS ≥ 2.1 and non-TI (observation where TI was not applied), the reasons for non-TI were descriptively analyzed.

In order to explore the relationship between ASDAS and TI, first the ASDAS at time of TI or non-TI was compared. Next, Receiver Operating Characteristic (ROC) curve analyses were conducted with the ASDAS scores on a continuous scale and TI as the outcome, using the Area Under the Curve (AUC) to assess the ability of the ASDAS to discriminate between TI and non-TI in clinical practice (interpretation: 0.5 < AUC < 0.7 = poor, 0.7 ≤ AUC < 0.8 = acceptable/fair, 0.8 ≤ AUC < 0.9 = excellent, ≥ 0.9 = outstanding) [17]. Next, the cut-off that discriminated best between TI and non-TI (“optimal cut-off”) was identified using the Youden index, maximizing the sum of the sensitivity and specificity minus one. Analyses were conducted using (1) all observations, (2) only using a randomly selected observation per patient per calendar year or (3) using only the first observation per patient during the whole period. The latter two analyses were done to achieve a balanced number of observations per patient by follow-up duration, considering that some patients have a more frequent follow-up than others and contribute more weight to the analysis without such adjustment. Some patients might have consistently high ASDAS scores over time due to other (non-axSpA) factors, such as pain sensitization, without any changes in treatment. These patients would otherwise be overrepresented in the analysis and possibly bias the results. The ROC analysis was also repeated by calendar year, to see if there was a trend in optimal ASDAS cut-off over time, potentially indicating uptake of the T2T recommendations.

In a sensitivity analysis, the selection of one observation per patient per calendar year was not done randomly, but based on ASDAS (observation with highest ASDAS was selected). In addition, four subgroup analyses were conducted. First, analyses were stratified by current bDMARD/tsDMARD use, by ever bDMARD/tsDMARD use and by number of prior bDMARD/tsDMARD, as the number of remaining treatment options (which is lower in those already exposed to bDMARD/tsDMARD) could affect the decision to intensify treatment. The results in the bDMARD/tsDMARD-naïve subgroup would also mirror the development process of the ASDAS, where the external reference was the physician decision to start a TNFi. Second, analyses were stratified by symptom duration, presence/absence of extra-musculoskeletal manifestations (EMMs: psoriasis, uveitis, inflammatory bowel disease [IBD]), axSpA subtype (radiographic vs. non-radiographic axSpA), sex and centre. For symptom duration, patients were stratified by ≤ 5 vs. >5 years and by ≤ 10 vs. >10 years symptom duration, as the number of observations that met the ASAS definition of early axSpA (< 2 years symptom duration [18]) was insufficient for analysis.

Missing data were not imputed. For all analyses, *p* < 0.05 was considered significant. Statistical analyses were conducted using Stata 14.2 (StataCorp, College Station, TX, USA).

## Results

### Population characteristics

Out of 405 patients with treatment data in SpA-Net, a total of 350 patients also had at least one ASDAS observation in the 2016–2022 period and could be included in the current study. The median duration of follow-up was 2.8 (IQR 1.0-4.4) years, during which patients contributed a median of 4 (IQR 2–9) observations, resulting in a total of 2,191 observations. At time of inclusion in the current study, the average age was 48.2 years (SD 14.3) and 152 participants were female (43.4%) (Table 1). The mean ASDAS score was 2.4 (SD 1.0), with most patients being in a state of (very) high disease activity (ASDAS ≥ 2.1: *n* = 213, 60.9%). About half were already on a bDMARD (*n* = 173, 49.4%) and none were on a tsDMARD.

**Table 1:** **Characteristics of the study population at time of inclusion in current study**

Characteristics	Total (*n* = 350)
Age, years	48.2 (14.3)
Female, n (%)	152 (43.4%)
High education, n (%)	101 (38.3%)
Employment, n (%)	140 (59.6%)
Current smoking, n (%)	56 (24.7%)
Symptom duration, years	18.1 (13.1)
HLA-B27 positive, n (%)	229 (72.7%)
History of uveitis, n (%)	91 (26.3%)
History of psoriasis, n (%)	55 (15.9%)
History of IBD, n (%)	50 (14.5%)
On NSAID, n (%)	159 (45.4%)
On csDMARD, n (%)	42 (12.0%)
On bDMARD, n (%)	173 (49.4%)
On tsDMARD, n (%)	0 (0.0%)
ASDAS	2.4 (1.0)
ASDAS ≥ 2.1, n (%)	213 (60.9%)
BASDAI (0–10)	4.4 (2.1)
CRP	6.9 (15.9)
Patient global (0–10)	4.4 (2.5)
Pain (0–10)	4.3 (2.4)
BASFI (0–10)	3.6 (2.4)
ASAS HI (0–17)	6.0 (3.4)
Physician global	2.3 (1.6)
TJC ≥ 1, n (%)	36 (22.6%)
SJC ≥ 1, n (%)	13 (8.2%)
Dactylitis count ≥ 1, n (%)	6 (3.1%)
Enthesitis count ≥ 1, n (%)	38 (20.0%)

ASAS HI, Assessment of SpondyloArthritis international Society Health Index; ASDAS, Axial Spondyloarthritis Disease Activity Score; BASDAI, Bath Ankylosing Spondylitis Disease Activity Index; BASFI, Bath Ankylosing Spondylitis Functional Index; bDMARD, biological disease-modifying antirheumatic drug; CRP, c-reactive protein; csDMARD, conventional synthetic disease-modifying antirheumatic drug; HLA-B27, human leucocyte antigen B27; IBD, inflammatory bowel disease; NSAID, nonsteroidal anti-inflammatory drug; SJC, swollen joint count; TJC, tender joint count; tsDMARD, targeted synthetic disease-modifying antirheumatic drug

### Treatment intensification

Out of the total of 2,191 observations, TI occurred in 243 instances (11.1%). The median time between the ASDAS measurement and TI was 2 (IQR 0–14) days. At the time of TI, patients were mostly using NSAIDs (*n* = 95, 39.1%) and/or bDMARDs (*n* = 102, 42.0%) (Table 2). In about one-third of TI events (*n* = 90, 37.0%), patients were bDMARD-naïve. Of the various types of TI, addition of a new medication was most frequent (*n* = 130, 53.5%), with most additions involving NSAIDs (*n* = 51, 39.2%) or bDMARDs (*n* = 51, 39.2%). Switching within the same drug class emerged as the second most common form of TI (*n* = 89, 36.6%), and most switches involved NSAIDs (*n* = 42, 37.8%) or bDMARDs (*n* = 51, 45.9%). By the end of follow-up, 231 patients (66.0%) had been exposed to a bDMARD or tsDMARD at some point during the study period.

**Table 2:** Characteristics of medication use and type of TI at time of TI event

Characteristic	All TI events^a^ (*n* = 243)
Medication use at time of TI^b, c^, n (%)	
NSAID	95 (39.1%)
csDMARD	25 (10.3%)
bDMARD	102 (42.0%)
tsDMARD	2 (0.8%)
Glucocorticoid	11 (4.5%)
No anti-inflammatory drug	73 (30.0%)
Type of TI^d^, n (%)	
Dose increase	2 (0.8%)
Frequency increase	0 (0.0%)
Switch	111 (45.7%)
Switch within same drug class	89 (36.6%)
Switch between drug classes	9 (3.7%)
Switch, other^e^	13 (5.3%)
Switched to:	
NSAID	42 (37.8%)
csDMARD	2 (1.8%)
bDMARD	51 (45.9%)
tsDMARD	2 (1.8%)
Glucocorticoid	0 (0.0%)
Multiple^f^	14 (12.6%)
Addition	130 (53.5%)
Drug added:	
NSAID	51 (39.2%)
csDMARD	8 (6.2%)
bDMARD	51 (39.2%)
tsDMARD	1 (0.8%)
Glucocorticoid	15 (11.5%)
Multiple#	4 (3.1%)

^a^Data for any kind of TI event, which could be multiple events per patient over time

^b^Medication that the patient was already on at time of decision to conduct TI

^c^Patients could be on multiple drug classes

^d^If a drug was stopped and another one was started, this was considered a ‘switch’. If a drug was started but no drug was stopped, this was considered an ‘addition’

^e^Other type of switch, for example if a drug was stopped and multiple drugs were added as part of TI (both within the same class and between different classes)

^f^If multiple drugs were initiated as part of TI

bDMARD, biological disease-modifying antirheumatic drug; csDMARD, conventional synthetic disease-modifying antirheumatic drug; NSAID, nonsteroidal anti-inflammatory drug; tsDMARD, targeted synthetic disease-modifying antirheumatic drug; TI, treatment intensification

### Relationship between ASDAS and TI

In the entire dataset (2,191 observations in 350 patients), the mean ASDAS at TI was significantly higher than at non-TI (3.0 vs. 2.3, *p* < 0.001), as was the proportion of patients with ASDAS ≥ 2.1 (83.5% versus 54.6%) (Table 3). Interestingly, even when disease activity was high (ASDAS ≥ 2.1), TI was applied in a minority of observations (*n* = 203/1,266, 16.0%); although this was still higher than when disease activity was low (ASDAS < 2.1; *n* = 40/925, 4.3%). In the group with an ASDAS ≥ 2.1, the mean ASDAS at time of TI (3.3) was higher than at time of non-TI (3.0).

**Table 3:** Relationship between ASDAS and TI

	All observations (*N* = 2,191 observations^a^)		One observation per patient per calendar year (*N* = 1,153 observations^a^)	
	TI	No TI	TI	No TI
N_obs_ (%)	243 (11.1%)^b^	1,948 (88.9%)^b^	131 (11.4%)^b^	1,022 (88.6%)^b^
ASDAS, mean (SD)^c^	3.0 (1.0)	2.3 (1.0)	3.0 (1.0)	2.3 (0.9)
ASDAS ≥ 2.1, n (%)^c^	203 (83.5%)	1,063 (54.6%)	109 (83.2%)	553 (54.1%)

Percentages represent proportion within TI or no TI subgroup, unless otherwise stated

^a^In 350 patients

^b^Percentage represents proportion of total number of observations

^c^Statistically significant difference in ASDAS and proportion with ASDAS ≥ 2.1 between TI and non-TI subgroups, when using all observations and when using one observation per patient per calendar year (all *p* < 0.001)

ASDAS, Axial Spondyloarthritis Disease Activity Score; TI, treatment intensification

**Fig. 1:**
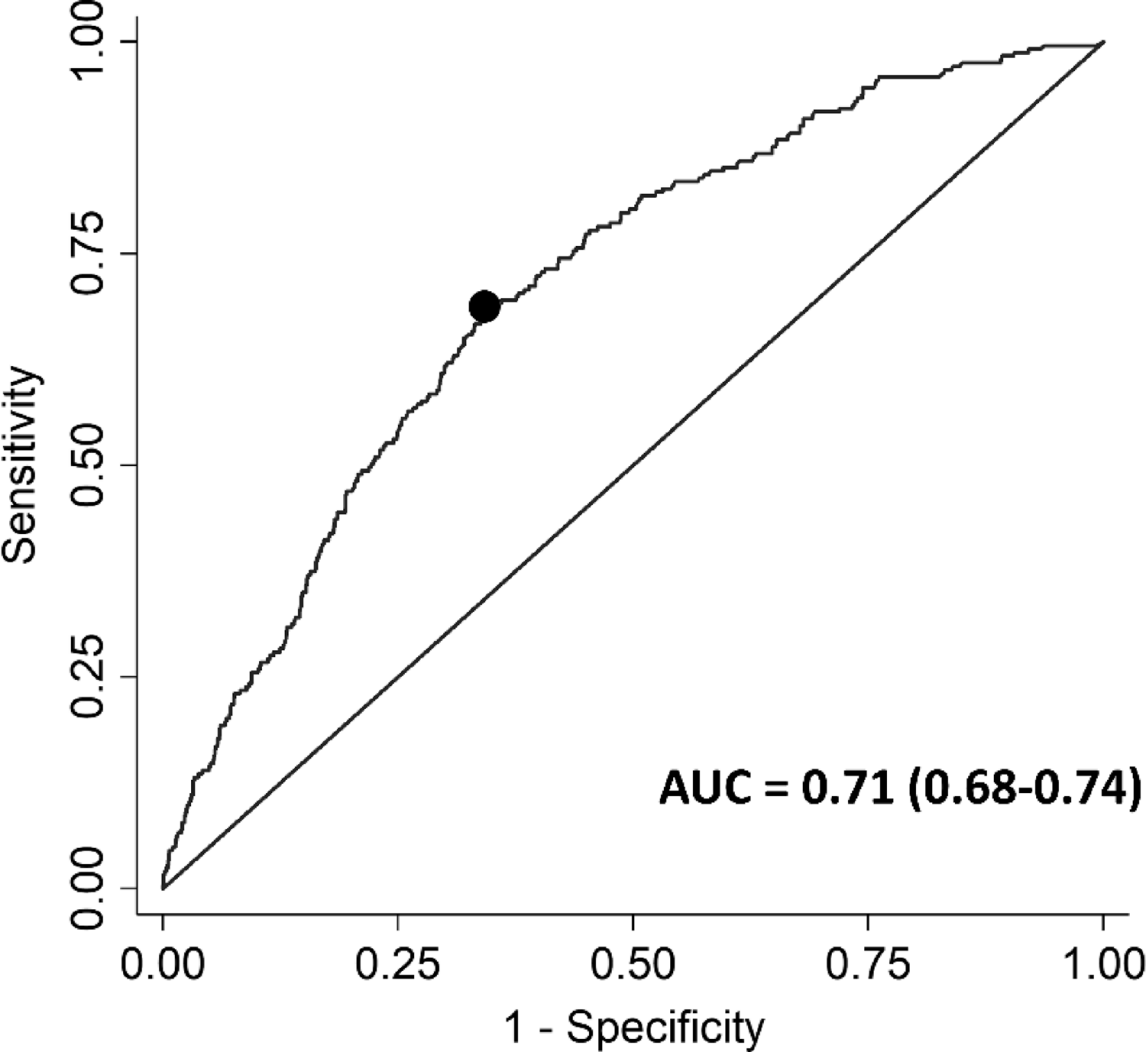
ROC curve of ASDAS and TI. Based on all ASDAS measurements (*n* = 2,191). The dot represents the optimal cut-off value (ASDAS 2.7), identified using the Youden index. ASDAS, Axial Spondyloarthritis Disease Activity Score; AUC, Area Under the Curve; ROC, Receiver Operating Characteristic; TI, treatment intensification

In the ROC analysis, the AUC was 0.71 (95% [CI] 0.68–0.74), indicating that the ASDAS had a fair ability to discriminate between TI and non-TI (Figure 1). An ASDAS cut-off value of 2.7 discriminated best between TI and non-TI (sensitivity 69%, specificity 66%). When only one observation per patient per calendar year was considered (1,153 observations in 350 patients), the mean ASDAS scores for TI and non-TI observations were similar to the primary analysis, as was the optimal ASDAS cut-off value (Table 3; AUC 0.75 [0.71–0.79], optimal cut-off = 2.7 [sensitivity 67%, specificity 68%]). Results were also in line when using only the first observation for each patient (optimal ASDAS cut-off: 2.7). Finally, for the individual calendar years (2016–2022), the optimal ASDAS cut-off value varied (range 2.3–2.9) without an apparent trend over time, but was always above the endorsed value of 2.1 (Table 4).

**Table 4:** ROC analysis of ASDAS cut-off and TI by year

	2016 (*n* = 59)	2017 (*n* = 186)	2018 (*n* = 230)	2019 (*n* = 226)	2020 (*n* = 153)	2021 (*n* = 161)	2022 (*n* = 138)
AUC (0.5-1.0)	0.81 (0.65–0.97)	0.76 (0.66–0.85)	0.71 (0.61–0.81)	0.64 (0.52–0.76)	0.71 (0.60–0.83)	0.68 (0.52–0.84)	0.76 (0.62–0.90)
Optimal cut-off^a^							
ASDAS	2.3	2.8	2.7	2.9	2.3	2.4	2.7
Sensitivity	1.00	0.67	0.75	0.52	0.89	0.83	0.73
Specificity	0.64	0.77	0.66	0.74	0.53	0.56	0.76

One observation per patient per year was used for analysis

^a^Based on Youden index (sum of sensitivity and specificity minus 1)

ASDAS, Axial Spondyloarthritis Disease Activity Score; AUC, Area Under the Curve; ROC, Receiver Operating Characteristic; TI, treatment intensification

### Sensitivity/subgroup analyses

If the observation with the highest ASDAS was chosen per patient per calendar year, the optimal ASDAS cut-off was similar (2.7) as in the main analysis. The subgroup analyses demonstrated that the results were mostly similar across various subgroups. The optimal ASDAS cut-off did not differ substantially by current use (2.9) or non-use (2.6) of bDMARD/tsDMARD, by ever being exposed to these drugs (2.7) or being naïve (2.6), or by number of prior bDMARD/tsDMARD (range 2.7–3.2 for 1 to ≥ 3 prior bDMARD/tsDMARD) (Online Resources 1–4). When only initiation of bDMARD/tsDMARD (and not NSAIDs/csDMARDs) was considered as TI in naïve patients, mirroring the original ASDAS development process (decision to initiate of TNFi), the optimal ASDAS cut-off was 2.8. The optimal ASDAS cut-off in patients with IBD, psoriasis or uveitis was similar as in patients without these EMMs (ASDAS cut-off range 2.6–2.8 versus 2.6–2.7 for EMM presence versus absence, respectively) (Online Resources 5 and 6). In the subgroup with a symptom duration of ≤ 5 years, the optimal cut-off was notably lower (2.1) than in the main analysis, although it should be noted that this subgroup was relatively small (*n* = 39 patients) (Online Resources 7 and 8). Finally, the optimal ASDAS cut-off value was similar in r-axSpA (2.7) and nr-axSpA (2.6), yet slightly higher in female (2.9) compared to male (2.6) patients, and in the MST hospital (3.1) compared to the MUMC + hospital (2.7) (Online Resources 9 and 10).

### Reasons for non-TI despite ASDAS ≥ 2.1

The electronic patient records of 35 patients with at least one ASDAS ≥ 2.1 without accompanying TI were manually checked. These patients contributed 246 observations to the main analysis, of which 26 observations (11%) had an ASDAS ≥ 2.1 with TI and 121 observations (49%) had an ASDAS ≥ 2.1 but no TI. The most frequently documented reasons for non-TI at these observations were that the rheumatologist considered the disease state to be acceptable (58 observations [48%] in 23 patients) or that there was another (non-axSpA) cause for the increased ASDAS score (30 observations [25%] in 15 patients), such as degenerative spinal changes or pain sensitization (Table 5). Rarely was it specifically the wish of the patient to not conduct TI despite active disease according to the rheumatologist. The mean ASDAS value was comparable across the different reasons (mean ASDAS range 2.4–2.8), except for “Patient does not want TI”, which had a notable higher score (3.6).

**Table 5:** Results from manual check of electronic patient record in random subset of patients

Reason for non-TI	Observations with no TI despite ASDAS ≥ 2.1 (*n* = 121 observations in 35 patients)
Rheumatologist considers state acceptable/stable	58 (48%)
Other (non-axSpA) cause of high ASDAS	30 (25%)
Expectant (wait-and-see)	6 (5%)
Awaiting diagnostic results	5 (4%)
Patient does not want TI	9 (7%)
Other^a^ or unknown	13 (11%)

Patients were eligible for the electronic patient record check if they had at least one observation of ASDAS ≥ 2.1 without accompanying TI. A random subset of patients was selected

^a^For example, if the ASDAS score was not yet known by the rheumatologist at the time of outpatient consultation (in some occasions, CRP testing and subsequent automated ASDAS calculation in SpA-Net took place after consultation)

ASDAS, Axial Spondyloarthritis Disease Activity Score; axSpA, axial spondyloarthritis; TI, treatment intensification

## Discussion

This observational study demonstrated that, while the ASDAS has a fair ability to discriminate between TI and non-TI, the ASDAS cut-off value associated with TI in clinical practice is higher than the endorsed cut-off value of 2.1. As expected, the mean ASDAS at TI was significantly higher than at non-TI. Additionally, even within the HDA group, only a minority (16%) received TI.

Our study was the first to investigate the ASDAS and TI in the current treatment era, where T2T has been more broadly accepted and even recommended in axSpA. Previously, a study in Turkey conducted in 2009 in patients with radiographic axSpA identified an ASDAS-CRP cut-off value of 2.4 to discriminate best between active disease (defined as requiring TNFi treatment) and inactive disease in routine practice [15]. These results are similar to ours, suggesting that the introduction of T2T in axSpA has thus far not impacted treatment decisions significantly.

The explanations for this discrepancy between the endorsed cut-off and the observed cut-off that is associated with TI in practice can be broadly divided into two categories. In the first category, rheumatologists do not use the ASDAS to adapt treatment. Our observation that the ASDAS has a fair ability to discriminate between TI and non-TI suggests that rheumatologists use this instrument (or the underlying construct, i.e. disease activity) only to a limited extent in their decision-making process, in line with previous research [9]. There could be several reasons for this. They might not be convinced of the benefits of T2T, or of using the ASDAS as the instrument to measure disease activity. The only trial that investigated T2T in axSpA (TICOSPA) failed to meet its primary endpoint [7]. In psoriatic arthritis, one of the barriers to implementation of T2T is the (lack of) belief of rheumatologists in the evidence supporting T2T; this might also apply to axSpA [19, 20]. On this line, the 2019 American College of Rheumatology/Spondylitis Association of America/Spondyloarthritis Research and Treatment Network (ACR/SAA/SPARTAN) management recommendations for axSpA do not endorse T2T due to lack of evidence supporting its benefit [6]. Likewise, in our retrospective analysis of patient records, we also observed that the rheumatologist considered the current state as acceptable despite an ASDAS of 2.1 or higher, suggesting that they do not necessarily target for a low ASDAS. Altogether, this suggests that there is a need for additional evidence on the (long-term) benefits of T2T management for patients with axSpA. Additional reasons for rheumatologists not using the ASDAS are that they are not capable of measuring the ASDAS – this did not apply in our study, as ASDAS was routinely collected – or are unaware that the ASDAS is the endorsed instrument to assess disease activity. Also, in some countries drug reimbursement does not depend on ASDAS but on other measures (such as BASDAI), which can influence use of the ASDAS.

In the second category, rheumatologists use the ASDAS for disease management, but do not change treatment when ASDAS is high. They might for example not be convinced of true disease activity. Comorbidities and other (non-axSpA) factors can lead to falsely elevated ASDAS scores, and preclude some patients from achieving and/or maintaining remission or LDA in practice [21, 22]. We saw evidence of this in our retrospective analysis using patient records: if no TI was conducted despite an ASDAS of 2.1 or higher, in a quarter of cases this was because the rheumatologist considered other (non-axSpA) factors as the cause of this increased ASDAS score. In certain patients, an ASDAS below 2.1 might not be a realistic target in clinical practice, and it is the question whether the endorsed cut-off of 2.1 should be mainly preserved for research purposes. This is further supported by the notion that patients with an elevated ASDAS often also consider themselves to be in an acceptable state [23, 24]. On this line, it is important to emphasize that it is undesirable to aim for TI in all situations where the ASDAS indicates high disease activity. Such a one-size-fits-all approach would likely lead to overtreatment and rapid cycling through treatment options, and ignores the patient’s context (going against recommendations) [1]. An additional reason for no treatment change despite high ASDAS is a lack of available treatment options. All treatment options could have failed or certain treatments could be contraindicated (e.g. due to comorbidity). Of note, in our study we did not see a clear discrepancy based on prior bDMARD/tsDMARD exposure. In light of the above considerations, our observation that rheumatologists often do not conduct TI when the ASDAS is above 2.1 might not be surprising. After all, treatment in axSpA should be individualized, and important patient factors (such as comorbidities) must be accounted for when making treatment decisions. However, such factors do not directly explain why we observed the optimal cut-off to be higher than the endorsed one.

We want to emphasize that our study does not indicate that we need to modify the ASDAS disease activity state cut-offs, and we do not advocate for such a change. Instead, we would argue that a multifaceted approach should be considered in the context of T2T in axSpA, focusing not only on disease inflammation as measured by ASDAS but also on disease impact. The dual target strategy, which includes both remission/LDA and overall disease impact, could be a beneficial framework [25]. However, more research is needed to explore the impact of such an approach in axSpA. Further research should also explore the reasons behind (non-)TI through qualitative or mixed-methods studies, capturing both the patient and the rheumatologist perspective. Investigating the role of the ASDAS (and T2T) in current practice and how rheumatologists arrive at treatment decisions is essential.

About one-third of TI events involved a switch to another drug within the same class. Likely, several factors played a role here. First, it is important to emphasize that we considered bDMARDs with different modes of action to be part of the same drug class in this study. For example, a switch from a TNFi to an IL-17 inhibitor was considered a “within-class” switch. This means that, in those already on a bDMARD, switches to another class mainly involve the use of tsDMARDs (i.e. JAK inhibitors), which have been relatively recently introduced in axSpA. Also, a majority of patients had already been exposed to a bDMARD at time of first observation, which limits their remaining treatment options. Finally, the best mode of action upon failure of the first bDMARD (often a TNFi) in axSpA is still unclear, which might result in switches within the same class (to another TNFi).

The current study’s main strength is the use of SpA-Net, which – as a daily practice registry in both academic and general hospital settings – represents current practice. Moreover, this study explores various types of pharmacological TI, unlike previous studies that primarily focused on TNFi. Of note, our choice to include any patient with a clinical diagnosis of axSpA (regardless of whether they met the ASAS classification criteria for axSpA or not [26]) was a deliberate one, as we wanted to follow daily practice – where patients are generally diagnosed and treated based on a clinical diagnosis – as much as possible. However, there are several limitations. First, we had only limited information on the reason that rheumatologists did (not) decide to conduct TI, and this was only explored in using retrospective analysis of free-text electronic patient records in SpA-Net. Second, we defined a state of disease activity based on the absolute ASDAS score and not on change in ASDAS (improvement). Patients on bDMARD/tsDMARD might have an ASDAS >2.1 but could still be responders if they achieved a clinically important or even major improvement in ASDAS; in such a situation, TI is often not conducted. However, in bDMARD/tsDMARD-naïve patients, our results were very similar. Third, ideally we would have also compared time points before and after introduction of major drug classes (e.g. IL-17 inhibitors), to assess the impact of drug availability. This was not possible, however, due to the delay between approval of new drugs and actual uptake in practice, as well as preferential use of certain drugs due to local policy. As an alternative, we explored the impact of drug availability on the individual level, by conducting sensitivity analyses in bDMARD/tsDMARD-naïve and exposed patients (with similar results). Finally, non-pharmacological treatments were not considered in this study, as they were not collected in SpA-Net. However, their effects on disease activity tend to be smaller and thus a less likely choice for patients with active disease.

In conclusion, the ASDAS cut-off associated with TI in axSpA patients in clinical practice exceeds the recommended cut-off. Additional research is required to uncover the process underlying treatment decisions, and to potentially reevaluate the current treatment strategies and definitions used in managing axSpA.

## Supplementary Information

Below is the link to the electronic supplementary material.

Supplementary Material 1

Supplementary Material 2

## Data Availability

The data that support the findings of this study are available from the corresponding author upon reasonable request.

## Declarations

### Note on related Congress abstracts and publications

This work has previously been presented as a poster presentation at the ACR Convergence 2023 congress (San Diego, United States), as an oral presentation at the EULAR 2024 European Congress of Rheumatology (Vienna, Austria) and as a poster presentation at the 14th International Congress on Spondyloarthritides (Ghent, Belgium). Abstracts have been published in Arthritis & Rheumatology (10.1002/art.42700, abstract number: 0517), Annals of the Rheumatic Diseases (https://10.1136/annrheumdis-2024-eular.2612, abstract number: OP0060), and Clinical and Experimental Rheumatology (10.55563/clinexprheumatol/7l5roe, abstract number: P106).

## References

[1] Ramiro S, Nikiphorou E, Sepriano A, Ortolan A, Webers C, Baraliakos X, Landewe RBM, Van den Bosch FE, Boteva B, Bremander A, Carron P, Ciurea A, van Gaalen FA, Geher P, Gensler L, Hermann J, de Hooge M, Husakova M, Kiltz U, Lopez-Medina C, Machado PM, Marzo-Ortega H, Molto A, Navarro-Compan V, Nissen MJ, Pimentel-Santos FM, Poddubnyy D, Proft F, Rudwaleit M, Telkman M, Zhao SS, Ziade N, van der Heijde D (2023) ASAS-EULAR recommendations for the management of axial spondyloarthritis: 2022 update. Ann Rheum Dis 82(1):19–34. 10.1136/ard-2022-223296 36270658 10.1136/ard-2022-223296

[2] Lukas C, Landewe R, Sieper J, Dougados M, Davis J, Braun J, van der Linden S, van der Heijde D, Assessment of SpondyloArthritis international S (2009) Development of an ASAS-endorsed disease activity score (ASDAS) in patients with ankylosing spondylitis. Ann Rheum Dis 68(1):18–24. 10.1136/ard.2008.094870 18625618 10.1136/ard.2008.094870

[3] Webers C, Ortolan A, Sepriano A, Falzon L, Baraliakos X, Landewe RBM, Ramiro S, van der Heijde D, Nikiphorou E (2023) Efficacy and safety of biological dmards: a systematic literature review informing the 2022 update of the ASAS-EULAR recommendations for the management of axial spondyloarthritis. Ann Rheum Dis 82(1):130–141. 10.1136/ard-2022-223298 36270657 10.1136/ard-2022-223298

[4] Ortolan A, Webers C, Sepriano A, Falzon L, Baraliakos X, Landewe RB, Ramiro S, van der Heijde D, Nikiphorou E (2023) Efficacy and safety of non-pharmacological and non-biological interventions: a systematic literature review informing the 2022 update of the ASAS/EULAR recommendations for the management of axial spondyloarthritis. Ann Rheum Dis 82(1):142–152. 10.1136/ard-2022-223297 36261247 10.1136/ard-2022-223297

[5] Smolen JS, Schols M, Braun J, Dougados M, FitzGerald O, Gladman DD, Kavanaugh A, Landewe R, Mease P, Sieper J, Stamm T, Wit M, Aletaha D, Baraliakos X, Betteridge N, Bosch FVD, Coates LC, Emery P, Gensler LS, Gossec L, Helliwell P, Jongkees M, Kvien TK, Inman RD, McInnes IB, Maccarone M, Machado PM, Molto A, Ogdie A, Poddubnyy D, Ritchlin C, Rudwaleit M, Tanew A, Thio B, Veale D, Vlam K, van der Heijde D (2018) Treating axial spondyloarthritis and peripheral spondyloarthritis, especially psoriatic arthritis, to target: 2017 update of recommendations by an international task force. Ann Rheum Dis 77(1):3–17. 10.1136/annrheumdis-2017-211734 28684559 10.1136/annrheumdis-2017-211734 PMC5754738

[6] Ward MM, Deodhar A, Gensler LS, Dubreuil M, Yu D, Khan MA, Haroon N, Borenstein D, Wang R, Biehl A, Fang MA, Louie G, Majithia V, Ng B, Bigham R, Pianin M, Shah AA, Sullivan N, Turgunbaev M, Oristaglio J, Turner A, Maksymowych WP, Caplan L (2019) 2019 update of the American college of Rheumatology/Spondylitis association of America/Spondyloarthritis research and treatment network recommendations for the treatment of ankylosing spondylitis and nonradiographic axial spondyloarthritis. Arthritis Rheumatol 71(10):1599–1613. 10.1002/art.41042 31436036 10.1002/art.41042 PMC6764882

[7] Molto A, Lopez-Medina C, Van den Bosch FE, Boonen A, Webers C, Dernis E, van Gaalen FA, Soubrier M, Claudepierre P, Baillet A, Starmans-Kool M, Spoorenberg A, Jacques P, Carron P, Joos R, Lenaerts J, Gossec L, Pouplin S, Ruyssen-Witrand A, Sparsa L, van Tubergen A, van der Heijde D, Dougados M (2021) Efficacy of a tight-control and treat-to-target strategy in axial spondyloarthritis: results of the open-label, pragmatic, cluster-randomised TICOSPA trial. Ann Rheum Dis 80(11):1436–1444. 10.1136/annrheumdis-2020-219585 33958325 10.1136/annrheumdis-2020-219585 PMC8522451

[8] Beckers E, Boonen A, Webers C, Ten Klooster P, Vonkeman H, Efde M, van Tubergen A (2022) Treat-to-target in axial spondyloarthritis: an observational study in daily practice. Rheumatology (Oxford) 61(4):1396–1407. 10.1093/rheumatology/keab516 34175950 10.1093/rheumatology/keab516 PMC8996808

[9] Webers C, Nezam El-Din R, Beckers E, Been M, Vonkeman HE, van Tubergen A (2025) Factors associated with treatment intensification in patients with axial spondyloarthritis and high disease activity in clinical practice. Rheumatology (Oxford) 64(1):91–98. 10.1093/rheumatology/kead634 38048595 10.1093/rheumatology/kead634 PMC11701311

[10] Bolt JW, Aalbers CJ, Walet L, van Mens LJJ, van Denderen C, van der Horst-Bruinsma I, van Baarsen LGM, Landewe R, van de Sande MGH (2024) Treatment decisions in axial spondyloarthritis daily clinical practice are more than treat-to-target. Rheumatology (Oxford) 63(1):34–40. 10.1093/rheumatology/kead155 37021937 10.1093/rheumatology/kead155 PMC10765143

[11] Machado PM, Landewe R, Heijde DV, Assessment of SpondyloArthritis international S (2018) Ankylosing spondylitis disease activity score (ASDAS): 2018 update of the nomenclature for disease activity States. Ann Rheum Dis 77(10):1539–1540. 10.1136/annrheumdis-2018-213184 29453216 10.1136/annrheumdis-2018-213184

[12] Machado P, Landewe R, Lie E, Kvien TK, Braun J, Baker D, van der Heijde D, Assessment of SpondyloArthritis international S (2011) Ankylosing spondylitis disease activity score (ASDAS): defining cut-off values for disease activity States and improvement scores. Ann Rheum Dis 70(1):47–53. 10.1136/ard.2010.138594 21068095 10.1136/ard.2010.138594

[13] Kilic E, Kilic G, Akgul O, Ozgocmen S (2015) Discriminant validity of the ankylosing spondylitis disease activity score (ASDAS) in patients with non-radiographic axial spondyloarthritis and ankylosing spondylitis: a cohort study. Rheumatol Int 35(6):981–989. 10.1007/s00296-014-3168-y 25366469 10.1007/s00296-014-3168-y

[14] Kilic G, Kilic E, Ozgocmen S (2017) Is there any gender-specific difference in the cut-off values of ankylosing spondylitis disease activity score in patients with axial spondyloarthritis? Int J Rheum Dis 20(9):1201–1211. 10.1111/1756-185X.12885 27309497 10.1111/1756-185X.12885

[15] Aydin SZ, Can M, Atagunduz P, Direskeneli H (2010) Active disease requiring TNF-alpha-antagonist therapy can be well discriminated with different ASDAS sets: a prospective, follow-up of disease activity assessment in ankylosing spondylitis. Clin Exp Rheumatol 28(5):752–755 20863448

[16] Webers C, Beckers E, Boonen A, van Eijk-Hustings Y, Vonkeman H, van de Laar M, van Tubergen A (2019) Development, usability and acceptability of an integrated eHealth system for spondyloarthritis in the Netherlands (SpA-Net). RMD Open 5(1):e000860. 10.1136/rmdopen-2018-000860 31168405 10.1136/rmdopen-2018-000860 PMC6525608

[17] Hosmer DW, Lemeshow S, Sturdivant RX (2013) Applied logistic regression. Wiley, Hoboken, New Jersey

[18] Navarro-Compan V, Benavent D, Capelusnik D, van der Heijde D, Landewe RB, Poddubnyy D, van Tubergen A, Baraliakos X, Van den Bosch FE, van Gaalen FA, Gensler L, Lopez-Medina C, Marzo-Ortega H, Molto A, Perez-Alamino R, Rudwaleit M, van de Sande M, Sengupta R, Weber U, Ramiro S (2024) ASAS consensus definition of early axial spondyloarthritis. Ann Rheum Dis 83(9):1093–1099. 10.1136/ard-2023-224232 37321799 10.1136/ard-2023-224232

[19] Dures E, Taylor J, Shepperd S, Mukherjee S, Robson J, Vlaev I, Walsh N, Coates LC (2020) Mixed methods study of clinicians’ perspectives on barriers to implementation of treat to target in psoriatic arthritis. Ann Rheum Dis 79(8):1031–1036. 10.1136/annrheumdis-2020-217301 32424031 10.1136/annrheumdis-2020-217301

[20] Smits ML, Webers C, van Dooren M, Mahler EAM, Vriezekolk JE, van Tubergen A (2025) Barriers and facilitators to treat-to-target in axial spondyloarthritis in clinical practice: a mixed methods study. Rheumatol Int 45(2):41. 10.1007/s00296-025-05795-6 39888406 10.1007/s00296-025-05795-6 PMC11785688

[21] Pina Vegas L, Sbidian E, Wendling D, Goupille P, Ferkal S, Le Corvoisier P, Ghaleh B, Luciani A, Claudepierre P (2022) Factors associated with remission at 5-year follow-up in recent-onset axial spondyloarthritis: results from the DESIR cohort. Rheumatology (Oxford) 61(4):1487–1495. 10.1093/rheumatology/keab565 34270707 10.1093/rheumatology/keab565 PMC8996779

[22] Benavent D, Franco-Gomez K, Plasencia-Rodriguez C, Novella-Navarro M, Bogas P, Nieto R, Monjo I, Nuno L, Villalba A, Peiteado D, Balsa A, Navarro-Compan V (2022) Achievement rate and predictive factors of the recommended therapeutical target in patients with axial spondyloarthritis who remain on biological therapy: a prospective cohort study in Spain. BMJ Open 12(4):e057850. 10.1136/bmjopen-2021-057850 35487753 10.1136/bmjopen-2021-057850 PMC9058765

[23] Godfrin-Valnet M, Prati C, Puyraveau M, Toussirot E, Letho-Gyselink H, Wendling D (2013) Evaluation of spondylarthritis activity by patients and physicians: ASDAS, BASDAI, PASS, and flares in 200 patients. Joint Bone Spine 80(4):393–398. 10.1016/j.jbspin.2013.01.003 23453478 10.1016/j.jbspin.2013.01.003

[24] Carbo M, Kampman A, Paap D, Wink F, Spoorenberg A, Arends S (2023) Pos0300 patient acceptable symptom state in relation to disease activity in patients with axial spondyloarthritis from a large Standard-of-Care cohort. Ann Rheum Dis 82(Suppl 1):392–393. 10.1136/annrheumdis-2023-eular.1008

[25] Ferreira RJO, Ndosi M, de Wit M, Santos EJF, Duarte C, Jacobs JWG, Machado PM, van der Heijde D, Gossec L, da Silva JAP (2019) Dual target strategy: a proposal to mitigate the risk of overtreatment and enhance patient satisfaction in rheumatoid arthritis. Ann Rheum Dis 78(10):e109. 10.1136/annrheumdis-2018-214199 30127056 10.1136/annrheumdis-2018-214199

[26] Rudwaleit M, van der Heijde D, Landewe R, Listing J, Akkoc N, Brandt J, Braun J, Chou CT, Collantes-Estevez E, Dougados M, Huang F, Gu J, Khan MA, Kirazli Y, Maksymowych WP, Mielants H, Sorensen IJ, Ozgocmen S, Roussou E, Valle-Onate R, Weber U, Wei J, Sieper J (2009) The development of assessment of spondyloarthritis international society classification criteria for axial spondyloarthritis (part II): validation and final selection. Ann Rheum Dis 68(6):777–783. 10.1136/ard.2009.108233 19297344 10.1136/ard.2009.108233

