# Supplementary material for: Which ASDAS cut-off corresponds best to treatment intensification in patients with axial spondyloarthritis in daily practice? A prospective study from a clinical registry
Source: Rheumatol Int. 2025 Oct 23;45(11):256. doi: 10.1007/s00296-025-06011-1 (PMC12549769; doi:10.1007/s00296-025-06011-1)
Supplement: Supplementary file 2 — Supplementary Material 2 [file 296_2025_6011_MOESM2_ESM.docx]

**Online Resource 1. Relationship between ASDAS and TI, stratified by current or ever b/tsDMARD exposure**

|  | **By current b/tsDMARD use** | | | | **By ever b/tsDMARD use** | | | |
| --- | --- | --- | --- | --- | --- | --- | --- | --- |
|  | **Currently on b/tsDMARD (N=738 observations)** | | **Currently not on b/tsDMARD (N=415 observations)** | | **b/tsDMARD exposed (N=810 observations)** | | **b/tsDMARD naive (N=343 observations)** | |
|  | **TI** | **No TI** | **TI** | **No TI** | **TI** | **No TI** | **TI** | **No TI** |
| N_obs_ | 52 | 686 | 79 | 336 | 75 | 735 | 56 | 287 |
| ASDAS, mean (SD) | 2.9 (1.1) | 2.2 (1.0) | 3.1 (1.0) | 2.3 (0.9) | 3.1 (1.1) | 2.2 (0.9) | 2.9 (1.0) | 2.3 (0.9) |
| ASDAS≥2.1, n (%) | 40 (76.9%) | 356 (51.9%) | 69 (87.3%) | 197 (58.6%) | 62 (82.7%) | 389 (52.9%) | 47 (83.9%) | 164 (57.1%) |

ASDAS, Axial Spondyloarthritis Disease Activity Score; b/tsDMARD, biological or targeted synthetic disease-modifying antirheumatic drug; TI, treatment intensification.

**Online Resource 2. ROC analysis of ASDAS ability to discriminate between TI and non-TI, stratified by current or ever b/tsDMARD exposure**

|  | **By current b/tsDMARD use** | | **By ever b/tsDMARD use** | |
| --- | --- | --- | --- | --- |
|  | **Currently on b/tsDMARD N=738 observations** | **Currently not on b/tsDMARD N=415 observations** | **b/tsDMARD exposed N=810 observations** | **b/tsDMARD naive N=343 observations** |
| AUC (0.5-1.0) | 0.70 (0.63-0.78) | 0.71 (0.64-0.77) | 0.74 (0.68-0.80) | 0.67 (0.60-0.75) |
| Optimal cut-off* |  |  |  |  |
| ASDAS | 2.9 | 2.6 | 2.7 | 2.6 |
| Sensitivity | 0.63 | 0.68 | 0.72 | 0.63 |
| Specificity | 0.76 | 0.65 | 0.70 | 0.66 |

*Based on Youden index (sum of sensitivity and specificity minus 1).

ASDAS, Axial Spondyloarthritis Disease Activity Score; AUC, Area Under the Curve; b/tsDMARD, biological or targeted synthetic disease-modifying antirheumatic drug; ROC, Receiver Operating Characteristic; TI, treatment intensification.

**Online Resource 3. Relationship between ASDAS and TI, stratified by number of prior b/tsDMARDs**

|  | **Number of prior b/tsDMARD** | | | | | | | |
| --- | --- | --- | --- | --- | --- | --- | --- | --- |
|  | **None (naïve) N=343 observations** | | **1 b/tsDMARD**  **N=394 observations** | | **2 b/tsDMARDs**  **N=177 observations** | | **≥3 b/tsDMARDs**  **N=239 observations** | |
|  | **TI** | **No TI** | **TI** | **No TI** | **TI** | **No TI** | **TI** | **No TI** |
| N_obs_ | 56 | 287 | 33 | 361 | 21 | 156 | 21 | 239 |
| ASDAS, mean (SD) | 2.9 (1.0) | 2.3 (0.9) | 3.0 (1.3) | 2.1 (1.0) | 3.0 (0.8) | 2.2 (0.8) | 3.3 (0.9) | 2.5 (0.9) |
| ASDAS≥2.1, n (%) | 47 (83.9%) | 164 (57.1%) | 24 (72.7%) | 161 (44.6%) | 18 (85.7%) | 85 (54.5%) | 20 (95.2%) | 143 (65.6%) |

ASDAS, Axial Spondyloarthritis Disease Activity Score; b/tsDMARD, biological or targeted disease-modifying antirheumatic drug; TI, treatment intensification.

**Online Resource 4. ROC analysis of ASDAS ability to discriminate between TI and non-TI, stratified by number of prior b/tsDMARDs**

|  | **By current b/tsDMARD use** | | | |
| --- | --- | --- | --- | --- |
|  | **None (naïve) N=343 observations** | **1 b/tsDMARD N=394 observations** | **2 b/tsDMARDs N=177 observations** | **≥3 b/tsDMARDs N=239 observations** |
| AUC (0.5-1.0) | 0.67 (0.60-0.75) | 0.72 (0.62-0.81) | 0.78 (0.66-0.90) | 0.76 (0.68-0.85) |
| Optimal cut-off* |  |  |  |  |
| ASDAS | 2.6 | 3.2 | 2.8 | 2.7 |
| Sensitivity | 0.63 | 0.48 | 0.71 | 0.95 |
| Specificity | 0.66 | 0.87 | 0.82 | 0.59 |

*Based on Youden index (sum of sensitivity and specificity minus 1).

ASDAS, Axial Spondyloarthritis Disease Activity Score; AUC, Area Under the Curve; b/tsDMARD, biological or targeted synthetic disease-modifying antirheumatic drug; ROC, Receiver Operating Characteristic; TI, treatment intensification.

**Online Resource 5. Relationship between ASDAS and TI, stratified by presence of extra-musculoskeletal manifestations**

|  | **IBD+ (N=156 observations)** | | **IBD- (N=990 observations)** | |
| --- | --- | --- | --- | --- |
|  | **TI** | **No TI** | **TI** | **No TI** |
| N_obs_ | 14 | 142 | 116 | 874 |
| ASDAS, mean (SD) | 3.6 (0.7) | 2.2 (0.8) | 2.9 (1.0) | 2.3 (1.0) |
| ASDAS≥2.1, n (%) | 14 (100.0%) | 76 (53.5%) | 94 (81.0%) | 473 (54.1%) |
|  |  |  |  |  |
|  | **Psoriasis+ (N=192 observations)** | | **Psoriasis- (N=954 observations)** | |
|  | **TI** | **No TI** | **TI** | **No TI** |
| N_obs_ | 25 | 167 | 105 | 849 |
| ASDAS, mean (SD) | 3.0 (0.9) | 2.4 (0.8) | 3.0 (1.0) | 2.2 (1.0) |
| ASDAS≥2.1, n (%) | 21 (84.0%) | 108 (64.7%) | 87 (82.9%) | 441 (51.9%) |
|  |  |  |  |  |
|  | **Uveitis+ (N=305 observations)** | | **Uveitis- (N=841 observations)** | |
|  | **TI** | **No TI** | **TI** | **No TI** |
| N_obs_ | 30 | 275 | 100 | 741 |
| ASDAS, mean (SD) | 3.1 (1.0) | 2.4 (0.9) | 3.0 (1.0) | 2.2 (1.0) |
| ASDAS≥2.1, n (%) | 27 (90.0%) | 158 (57.4%) | 81 (81.0%) | 391 (52.8%) |

ASDAS, Axial Spondyloarthritis Disease Activity Score; IBD, inflammatory bowel disease; TI, treatment intensification.

**Online Resource 6. ROC analysis of ASDAS ability to discriminate between TI and non-TI, stratified by presence of extra-musculoskeletal manifestations**

|  | **Stratified by IBD** | | **Stratified by psoriasis** | | **Stratified by uveitis** | |
| --- | --- | --- | --- | --- | --- | --- |
|  | **IBD+**  **N=156 observations** | **IBD-**  **N=990 observations** | **Psoriasis+**  **N=192 observations** | **Psoriasis-**  **N=954 observations** | **Uveitis+**  **N=305 observations** | **Uveitis-**  **N=841 observations** |
| AUC (0.5-1.0) | 0.93 (0.89-0.98) | 0.69 (0.64-0.74) | 0.70 (0.58-0.81) | 0.71 (0.66-0.77) | 0.71 (0.62-0.80) | 0.71 (0.66-0.77) |
| Optimal cut-off* |  |  |  |  |  |  |
| ASDAS | 2.8 | 2.6 | 2.7 | 2.7 | 2.6 | 2.7 |
| Sensitivity | 1.00 | 0.64 | 0.72 | 0.65 | 0.70 | 0.67 |
| Specificity | 0.76 | 0.67 | 0.67 | 0.70 | 0.65 | 0.69 |

*Based on Youden index (sum of sensitivity and specificity minus 1).

ASDAS, Axial Spondyloarthritis Disease Activity Score; AUC, Area Under the Curve; IBD, inflammatory bowel disease; ROC, Receiver Operating Characteristic; TI, treatment intensification.

**Online Resource 7. Relationship between ASDAS and TI, stratified by symptom duration**

|  | **By symptom duration, ≤5 vs >5 years** | | | | **By symptom duration, ≤10 vs >10 years** | | | |
| --- | --- | --- | --- | --- | --- | --- | --- | --- |
|  | **≤5 years**  **N=81 observations** | | **>5 years**  **N=878 observations** | | **≤10 years**  **N=240 observations** | | **>10 years**  **N=719 observations** | |
|  | **TI** | **No TI** | **TI** | **No TI** | **TI** | **No TI** | **TI** | **No TI** |
| N_obs_ | 15 | 66 | 105 | 773 | 44 | 196 | 76 | 643 |
| ASDAS, mean (SD) | 3.2 (1.2) | 2.3 (1.1) | 2.9 (0.9) | 2.2 (0.9) | 3.1 (1.1) | 2.3 (1.0) | 2.9 (0.9) | 2.2 (0.9) |
| ASDAS≥2.1, n (%) | 14 (18.5%) | 34 (51.5%) | 85 (81.0%) | 416 (53.8%) | 38 (86.4%) | 99 (50.5%) | 61 (80.3%) | 351 (54.6%) |

ASDAS, Axial Spondyloarthritis Disease Activity Score; TI, treatment intensification.

**Online Resource 8. ROC analysis of ASDAS ability to discriminate between TI and non-TI, stratified by symptom duration**

|  | **Symptom duration ≤5 vs >5 years** | | **Symptom duration ≤10 vs >10 years** | |
| --- | --- | --- | --- | --- |
|  | **≤5 years**  **N=81 observations** | **>5 years**  **N=878 observations** | **≤10 years**  **N=240 observations** | **>10 years**  **N=719 observations** |
| AUC (0.5-1.0) | 0.71 (0.57-0.84) | 0.70 (0.65-0.75) | 0.71 (0.63-0.79) | 0.69 (0.63-0.76) |
| Optimal cut-off* |  |  |  |  |
| ASDAS | 2.1 | 2.6 | 2.7 | 2.9 |
| Sensitivity | 0.93 | 0.68 | 0.70 | 0.57 |
| Specificity | 0.50 | 0.67 | 0.66 | 0.76 |

*Based on Youden index (sum of sensitivity and specificity minus 1).

ASDAS, Axial Spondyloarthritis Disease Activity Score; AUC, Area Under the Curve; ROC, Receiver Operating Characteristic; TI, treatment intensification.

**Online Resource 9. Relationship between ASDAS and TI, stratified by axSpA subtype, sex and by centre**

|  | **By axSpA subtype** | | | | | | **By sex** | | | | | | **By centre** | | | | | |
| --- | --- | --- | --- | --- | --- | --- | --- | --- | --- | --- | --- | --- | --- | --- | --- | --- | --- | --- |
|  | **r-axSpA**  **N=722 observations** | | | **nr-axSpA**  **N=424 observations** | | | **Female**  **N=507 observations** | | | **Male**  **N=646 observations** | | | **MUMC+**  **N=701 observations** | | | **MST**  **N=452 observations** | | |
|  | **TI** | **No TI** | **TI** | | **No TI** | **TI** | | **No TI** | **TI** | | **No TI** | **TI** | | **No TI** | **TI** | | **No TI** |  |
| N_obs_ | 60 | 662 | 70 | | 354 | 73 | | 434 | 58 | | 588 | 91 | | 610 | 40 | | 412 |  |
| ASDAS, mean (SD) | 3.2 (1.1) | 2.2 (1.0) | 2.9 (0.9) | | 2.3 (0.9) | 3.1 (1.0) | | 2.4 (0.8) | 2.9 (1.0) | | 2.1 (1.0) | 2.8 (0.9) | | 2.2 (0.9) | 3.4 (1.1) | | 2.3 (1.1) |  |
| ASDAS≥2.1, n (%) | 51 (85.0%) | 340 (51.4%) | 57 (81.4%) | | 209 (59.0%) | 64 (87.7%) | | 283 (65.2%) | 45 (77.6%) | | 270 (45.9%) | 73 (80.2%) | | 325 (53.3%) | 36 (90.0%) | | 228 (55.3%) |  |

ASDAS, Axial Spondyloarthritis Disease Activity Score; MST, Medisch Spectrum Twente; MUMC+, Maastricht University Medical Centre; nr-axSpA, non-radiographic axial spondyloarthritis; r-axSpA, radiographic axial spondyloarthritis; TI, treatment intensification.

**Online Resource 10. ROC analysis of ASDAS ability to discriminate between TI and non-TI, stratified by axSpA subtype, sex and by centre**

|  | **By axSpA subtype** | | **By sex** | | **By centre** | |
| --- | --- | --- | --- | --- | --- | --- |
|  | **r-axSpA**  **N=722 observations** | **nr-axSpA**  **N=424 observations** | **Female**  **N=507 observations** | **Male**  **N=646 observations** | **MUMC+**  **N=701 observations** | **MST**  **N=452 observations** |
| AUC (0.5-1.0) | 0.74 (0.68-0.81) | 0.67 (0.61-0.74) | 0.71 (0.64-0.77) | 0.71 (0.65-0.78) | 0.70 (0.64-0.75) | 0.76 (0.69-0.84) |
| Optimal cut-off* |  |  |  |  |  |  |
| ASDAS | 2.7 | 2.6 | 2.9 | 2.6 | 2.7 | 3.1 |
| Sensitivity | 0.67 | 0.67 | 0.63 | 0.64 | 0.64 | 0.65 |
| Specificity | 0.72 | 0.64 | 0.72 | 0.71 | 0.71 | 0.78 |

*Based on Youden index (sum of sensitivity and specificity minus 1).

ASDAS, Axial Spondyloarthritis Disease Activity Score; AUC, Area Under the Curve; MST, Medisch Spectrum Twente; MUMC+, Maastricht University Medical Centre; nr-axSpA, non-radiographic axial spondyloarthritis; r-axSpA, radiographic axial spondyloarthritis; ROC, Receiver Operating Characteristic; TI, treatment intensification.
